# The global distribution of *Paragonimus* species

**DOI:** 10.1186/s40249-026-01428-7

**Published:** 2026-02-27

**Authors:** Yuan-Biao Lu, Kai Hu, Mei-Lin Mao, Lin-Bo Shi, Chun-Chao Zhu, Jie-Xin Zou

**Affiliations:** 1 Research Laboratory of Freshwater Crustacean Decapoda and Paragonimus, School of Basic Medical Sciences, Jiangxi Medical College, Nanchang University, Nanchang, 330031 Jiangxi Province China; 2 Department of Parasitology, School of Basic Medical Sciences, Jiangxi Medical College, Nanchang University, Nanchang, 330031 Jiangxi Province China; 3 Provincial Key Laboratory for Drug Targeting and Drug Screening, Jiangxi Medical College, Nanchang University, Nanchang, 330031 China

**Keywords:** *Paragonimus*, Paragonimiasis, Lung fluke, Distribution, Host

## Abstract

**Background:**

Paragonimiasis, a foodborne zoonotic disease caused by *Paragonimus* (lung fluke) species, is prevalent mainly in tropical and subtropical regions. It is estimated that 23 million people are infected worldwide. Many reviews have been published in recent years, but very few reviews focused on distribution have been published. Here, we performed a review to map the global distribution of *Paragonimus* species.

**Methods:**

We systematically searched Google Scholar, Web of Science, PubMed, ScienceDirect, African Journals Online, the Chinese National Knowledge Infrastructure (CNKI), Wanfang Database, and Chongqing VIP Chinese Science and Technology Journal Database to identify studies and case reports documenting the occurrences of *Paragonimu*s species (OPSs) and cases of paragonimiasis. Studies were included only if both the OPSs and the geographical locations of *Paragonimus* species occurrence had been verified in the relevant research. Subsequently, we mapped the OPSs based on data extracted from the eligible included studies.

**Results:**

We mapped the global distribution of *Paragonimus* species and cases caused by them. *Paragonimus* species are distributed in Asia, Africa, and the Americas, among which no species are distributed across continents. Eight species can infect humans: *P. westermani* (East Asia, the Far East, Southeast Asia, India, Sri Lanka, probably Nepal, New Guinea), *P. skrjabini* (mainly distributed in China and Japan), *P. heterotremus* (the Indochina Peninsula and southwestern border regions of China), *P. kellicotti* (USA and Canada), *P. mexicanus* (Latin America), *P. africanus*, *P. uterobilateralis*, and *P. gondwanensis* (West and Central Africa). This study suggests that *Paragonimus* species are distributed from 12° S to 50° N globally, except four “outliers” in South Africa (approximately 30° S).

**Conclusions:**

Globally, *Paragonimus* species and paragonimiasis cases exhibit an imbalanced distribution across hemispheres, continents, and countries. In many “suitable habitats”, where infection by *Paragonimus* may be expected, few or no OPSs have been reported. Epidemiological and other studies are encouraged in these regions. This study will support the further refinement of paragonimiasis surveillance and response measures by global disease control authorities, thereby advancing public health.

**Graphical Abstract:**

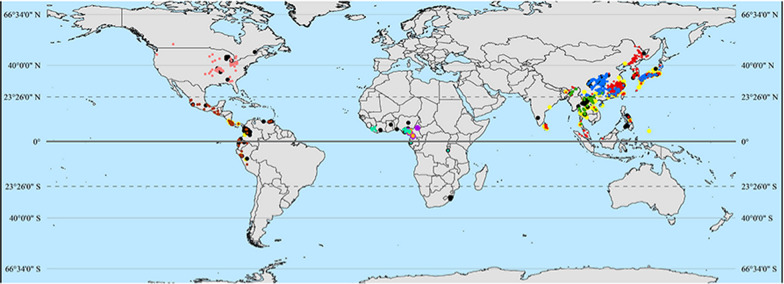

**Supplementary Information:**

The online version contains supplementary material available at 10.1186/s40249-026-01428-7.

## Background

**Fig. 1:**
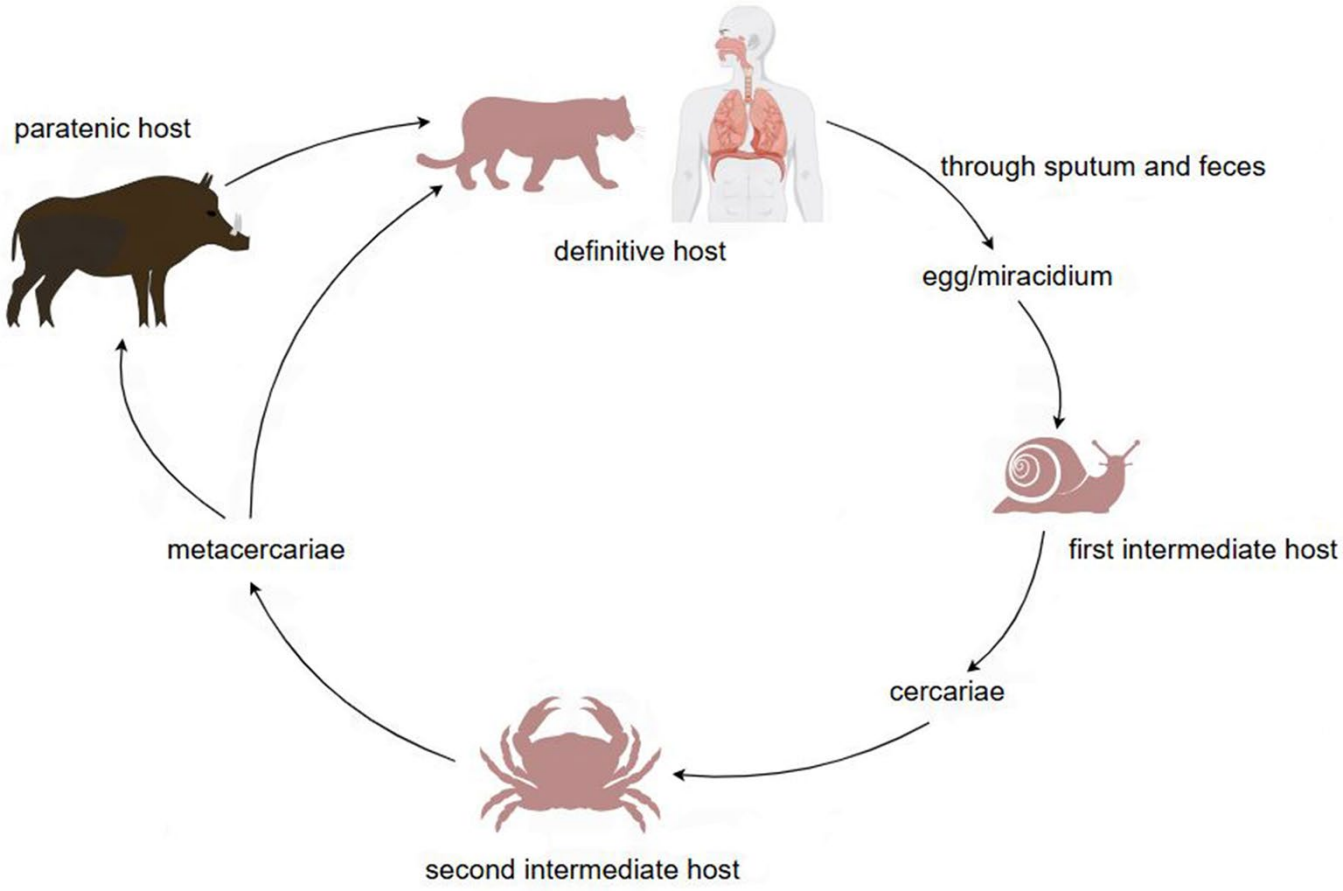
Life cycle typical of *Paragonimus* species including names of life-cycle stages in the corresponding hosts

Paragonimiasis, a neglected tropical disease [1], is a foodborne zoonotic disease caused by *Paragonimus* (lung fluke) species. It is broadly distributed in tropical and subtropical regions, including Asia, Africa and the Americas [2–5]. It was estimated that 23 million people are infected worldwide, mostly in Asia [6]. *Paragonimus* species are hermaphroditic trematodes with complex life cycles and at least three types of hosts: first intermediate hosts, second intermediate hosts and definitive hosts [2, 7]. Eggs of *Paragonimus* species produced by adult worms infecting definitive hosts can enter water through feces and sputum. Eggs develop into miracidia in water at suitable temperatures (Fig. 1). When miracidia meet the first intermediate hosts (usually freshwater snails), they enter the snails and give rise eventually to cercariae. Cercariae released by snails infect second intermediate hosts (usually freshwater crabs and crayfish) and develop into metacercariae in crustaceans (Fig. 1). Animals (definitive hosts) can be infected by ingesting crustaceans, and then metacercariae can develop into adults. Paratenic hosts are optionally involved in the life cycle, because sometimes metacercariae do not develop into adults but remain as juveniles in the tissues of the hosts [2, 3, 5, 7]. Among the more than 50 species worldwide, eight can infect humans [5]: *Paragonimus westermani* (Asia), *P. skrjabini* (Asia), *P. heterotremus* (Asia), *P. africanus* (Africa), *P. uterobilateralis* (Africa), *P. gondwanensis* (Africa), *P. kellicotti* (North America), and *P. mexicanus* (Central and South America). The human cases of infection with *P. gondwanensis* are based on parasite eggs in a couple of humans only, so that there is some uncertainty about this diagnosis. Humans generally get infected by consuming raw or undercooked crustaceans. Sometimes, infection in humans occurs through the consumption of paratenic hosts (such as case reports of the consumption of wild boars in Japan) [7–10]. In parts of Asia, paragonimiasis is more common due to pickling or eating raw or uncooked freshwater crabs, such as the “drunk crab” in China and the “kejiang” in the Republic of Korea [11–14]. In Africa, however, diseases caused by foodborne trematodes are rare because the thorough cooking of most foods, a tradition in Africa, will kill metacercariae [15, 16]. Among *Paragonimus* species that typically cause pulmonary disease, some worms may cause pleural disease, or stray beyond the thoracic cavity to cause ectopic paragonimiasis [17], which can affect almost any part of the body. In areas endemic for tuberculosis (TB), patients initially misdiagnosed with drug-resistant TB were found to be infected with paragonimiasis, confirming that the region is also endemic for paragonimiasis [18].

Traditionally, the taxonomy of *Paragonimus* species was based on the morphology of adult worms and the form of metacercariae [5]. However, for many *Paragonimus* species, there are too few morphological features of the adult stage to distinguish between species, and the morphological characteristics of the metacercariae stage are considered unreliable [19, 20]. With the development of sequencing technology, molecular data have become popular in the areas of taxonomy and phylogeny. The addition of molecular data has helped solve some taxonomy problems. For example, the systematic position of *P. caliensis* Little, 1968, was determined via the use of molecular data [21]. However, the application of molecular data simultaneously adds to the complexity of taxonomy [5]. Whether the challenge of taxonomic classification becomes simpler or more complex hinges on the degree to which molecular phylogenetic information aligns with the morphological characteristics of *Paragonimus* species.

### Species

We here present the world register of *Paragonimus* species based on studies by Blair et al. 1999, Yoshida et al. 2019, and Zhou et al. 2021 [2–4]. In the twenty-first century, five new species of *Paragonimus* were reported: *P. vietnamensis* from Vietnam (Asia), *P. pseudoheterotremus* from Thailand (Asia), *P. sheni* from China (Asia), and *P. gondwanensis* and *P. kerberti* from Cameroon (Africa) (Table 1). Two species of the genus *Euparagonimus* were included in Table 1 in this study. The reason was that multiple studies have shown that *Euparagonimus* is highly similar to species of the *Paragonimus* in terms of phylogenetic relationships and both should belong to the genus *Paragonimus*, despite some differences between them in morphology and life cycle [5, 22]*.* The two *Euparagonimus* species were distributed only in southeastern China. Although there are 57 nominal *Paragonimus* species worldwide, no species are distributed across continents (the Americas is regarded as a whole). More information about the taxonomy, synonyms and distribution of *Paragonimus* species is provided in Table 1. In the field of public health research, there are a total of 8 human-infecting species globally, with three in Asia, two in the Americas and three in Africa. A brief introduction to the eight species follows.

**Table 1:** The world register of *Paragonimus* species

No	Species	Synonyms	Second hosts (family)	Distribution
1	*P. bangkokensis* (Miyazaki and Vajrasthira, 1967)	No synonym	Potamidae	Thailand, China, Laos, Vietnam
2	*P. cheni* (Hu, 1963)	No synonym	Potamidae	China
3	*P. compactus* (Cobbold, 1859)	No synonym	Not sure	India, Sri Lanka
4	*P. fukienensis* (Tang and Tang, 1962)	No synonym	Potamidae, Gecarcinucidae	China
5	*P. harinasutai* (Miyazaki and Vajrasthira, 1968)	No synonym	Potamidae, Gecarcinucidae	Thailand, China, Laos, Vietnam
6	*P. heterorchis* (Zhou et al., 1982)	No synonym	Potamidae	China
7	*P. heterotremus* (Chen and Hsia, 1964)	*P. tuanshanensis* (Chung et al., 1964)	Potamidae, Gecarcinucidae	China, Thailand, Laos, Vietnam, India
8	*P. jiangsuensis* (Cao et al., 1983)	*P. xiangshanensis* (He et al., 1995)	Potamidae	China
9	*P. macrorchis* (Chen, 1962)	No synonym	Gecarcinucidae, Potamidae	China, Sri Lanka, Thailand
10	*P. microchis* (Hsia et al., 1978)	No synonym	Potamidae	China
11	*P. minqinensis* (Li and Chen, 1983)	No synonym	Potamidae	China
12	*P. mungoi* (Mishra et al., 1976)*	No synonym	Not sure	India
13	*P. ohirai* (Miyazaki, 1939)	*P. sadoensis* (Miyazaki et al., 1968) *P. iloktsuenensis* (Chen, 1940)	Potamidae, Sesarmidae, Varunidae	Japan, China, the Republic of Korea
14	*P. paishuihoensis* (Tsao and Chung, 1965)	*P. divergens* (Liu et al., 1980)	Potamidae	China, Laos, Thailand
15	*P. pantheri* (Mishra and Patal, 1976)*	No synonym	Not sure	India
16	*P. proliferus* (Hsia and Chen, 1964)	*P. menglaensis* (Chung et al., 1964) *P. hokuoensis* (Ho et al., 1965)	Potamidae	China, Vietnam
17	*P. siamensis* (Miyazaki and Wykoff, 1965)	No synonym	Gecarcinucidae, Potamidae	Thailand, Sri Lanka
18	*P. skrjabini* (Chen, 1959)	*P. hueitungensis* (Chung et al., 1975) *P. miyazakii* (Kamo et al., 1961) *P. szechuanensis* (Chung and Tsao, 1962) *P. veocularis* (Chen and Li, 1979)	Potamidae	China, Japan, Thailand, India, Vietnam
19	*P. sheni* (Shan, Lin et al., 2009.)	No synonym	Potamidae	China
20	*P. taipingini* (Kurochkin, 1987)**	No synonym	Not sure	China
21	*P. westermani* (Kerbert, 1878)	*P. asymmetricus* (Chen, 1977) *P. edwardsi* (Gulati, 1926) *P. filipinus* (Miyazaki, 1978) *P. macacae* (Sandosham, 1953) *P. philippinensis* (Ito et al., 1978) *P. pulmonalis* (Baelz, 1880) *P. ringeri* (Cobbold, 1880)	Potamidae, Gecarcinucidae, Cambaroididae, Varunidae	India, Nepal, Thailand, the Philippines, Indonesia, Papua New Guinea, Myanmar, Vietnam, Cambodia, Laos, the Republic of Korea, China, Japan, Far East of Russia
22	*P. yunnanensis* (Ho et al., 1959)	No synonym	Potamidae	China
23	*P. vietnamensis* (Doanh et al., 2007)	No synonym	Potamidae	Vietnam
24	*P. pseudoheterotremus* (Waikagul, 2007)	No synonym	Potamidae	Thailand
25	*P. africanus* Voelker and Vogel, 1965	No synonym	Potamonautidae	Cameroon, Nigeria, Gabon, Equatorial Guinea, Ivory Coast
26	*P. kerberti* Bayssade-dufour et al., 2015	No synonym	Potamonautidae	Cameroon
27	*P. gondwanensis* Bayssade-dufour et al., 2014	No synonym	Potamonautidae	Cameroon, Ivory Coast
28	*P. uterobilateralis* Voelker and Vogel, 1965	No synonym	Potamonautidae	Cameroon, Nigeria, Liberia, Guinea, Gabon
29	*P. amazonicus* Miyazaki, Grados and Uyema, 1973	No synonym	Pseudothelphusidae	Peru
30	*P. caliensis Little*, 1968	No synonym	Pseudothelphusidae	Colombia, Peru, Panama, Mexico, Costa Rica
31	*P. inca* Miyazaki, Mazabel, Grados and Uyema, 1975	No synonym	Pseudothelphusidae, Trichodactylidae	Peru
32	*P. kellicotti* Ward, 1908	No synonym	Cambaroididae	USA, Canada
33	*P. mexicanus* Miyazaki and Ishii, 1968	*P. ecuadoriensis* Voelker and Arzube, 1979 *P. peruvianus* Miyazaki et al., 1969	Pseudothelphusidae, Trichodactylidae	Mexico, Costa Rica, Panama, Guatemala, Ecuador, Peru
34	*P. napensis* Amunarriz, 1991	No synonym	Not sure	Ecuador
35	*P. rudis* (Diesing, 1850) Stiles and Hassall, 1900**	No synonym	Not sure	Brazil
36	*Euparagonimus cenocopiosus* (Chen, 1962)	No synonym	Potamidae, Gecarcinucidae	China
37	*Euparagonimus hongzesiensis* (Fu et al., 1990)	No synonym	Not sure	China

The synonyms, second hosts and country-level distributions of *Paragonimus* species are also shown

^*^: Nomen nudum

^**^: Species inquirenda

#### Paragonimus westermani

The first reported *Paragonimus* species was described from the lungs of the otter *Pteronura braziliensis* (Gmelin, 1788) in Brazil [2]. In 1878, Kerbert described a new species, *Distomum westermani,* from a Bengal tiger at the Amsterdam zoological gardens in the Netherlands [23]. The following year, Dr. Ringer detected a species of parasite, *Distomum ringeri* (Cobbold, 1880), in the body of a Portuguese sailor from Taiwan, China, which was the first time that human paragonimiasis was reported [24]. Shortly thereafter, Baelz reported a species, *D. pulmonalis* (Baelz, 1883), in Japan [24]. In 1899, Braun split the genus *Distomum* to many genera*,* one of which is *Paragonimus*, and the name “Paragonimus” is still used [24]. The referred two species above (*D. ringeri* and *D. pulmonalis*) were regarded as synonyms of *Paragonimus westermani*. In later studies, many new species, such as *P. asymmetricus* (Chen, 1977), *P. edwardsi* (Gulati, 1926) and *P. filipinus* (Miyazaki, 1978), were reported and regarded as synonyms of *P. westermani* [2, 25–30].

#### Paragonimus skrjabini

Chen first briefly described *P. skrjabini* from the lungs of the viverrid *Paguma larvata*, which was purchased from markets in Guangzhou (a city in southern China) in 1959, and more morphological descriptions were provided for the following year [27]. In 1962, Chung and Tsao described *P. szechuanensis* from cats in Sichuan Province [31, 32]. However, Chen compared this species with *P. skrjabini* in terms of the morphological aspects of the cuticular spines, the shape and size of the eggs, the body proportions of the adults and the species of the hosts and reported that *P. szechuanensis* is the same as *P. skrjabini* [33]. In 2003, Cui et al. collected five types of geographical strains of *P. skrjabini* from different provinces in China and verified that *P. szechuanensis* is a synonym of *P. skrjabini* on the basis of the morphology and phylogeny of different geographical strains [34, 35]. *Paragonimus hueitungensis*, *P. veocularis* and *P. miyazakii* are also regarded as synonyms of *P. skrjabini* [2, 27, 28, 36, 37] (Table 1).

#### Paragonimus heterotremus

*Paragonimus heterotremus* was first reported in Guangxi Zhuang Autonomous Region, China by Chen and Hsia in 1964, but specific collection locations, collection times and collectors were not provided in this study [38]. In the same year, Chung et al. described *P. tuanshanensis* from Xishuangbanna Dai Autonomous Prefecture, Yunnan Province [39]. According to Chen in 1965, *P. tuanshanensis* is regarded as a synonym of *P. heterotremus* [40]. Li et al. performed a detailed comparison of the morphology of the two species and considered the two to be the same species [27].

#### Paragonimus kellicotti

The discovery of *P. kellicotti* was almost simultaneously reported by Ward and Kellicott in 1894 [41]. After the description of infection of this species in a cat that was found in Michigan, United States by Ward, Kellicott described the infection in a dog in Columbus, Ohio, the United States later that year [42]. However, Ward identified the species as *Distoma westermani* at that time [43]. Subsequently, Ward and Hirsch determined that the North American species was a distinct *Paragonimus* species [44], which they named *P. kellicotti*. *P. kellicotti* is the only *Paragonimus* species endemic to North America [24]. Paragonimiasis caused by *P. kellicotti* is rare in humans but is usually observed in wild and domestic animals [24, 45].

#### Paragonimus mexicanus

The species was described from Colima, Mexico, by Miyazaki and Ishii in 1968 [46]. *Paragonimus ecuadoriensis* and *P. peruvianus* were described from coastal Ecuador in 1979 and from Peru in 1969, respectively. These two species were regarded as synonyms of *P. mexicanus* [47] by Miyazaki et al. However, López-Caballero considered that *P. mexicanus* is a species complex [48]. Tongu suggested that the other six species in Latin America are *P. mexicanus* [49]. Usually, only *P. ecuadoriensis* and *P. peruvianus* are regarded as synonyms of *P. mexicanus* [2]. Studies on the taxonomy of *Paragonimus* species in Latin America are ongoing [50, 51]. *P. mexicanus* is distributed in all countries in Latin America where *Paragonimus* species are endemic [2, 5, 51].

#### Paragonimus africanus

*Paragonimus africanus* was described from Lower Bakossi, West Cameroon, by Voelker and Vogel in 1965 [52]. The species is distributed mainly in West Africa [52, 53]. The species richness of *Paragonimus* species in Africa is low (only four), and none of the species has synonyms. Paragonimiasis caused by this species is usually pulmonary [5].

#### Paragonimus uterobilateralis

This species was reported together with *P. africanus* from Cameroon in the same study [52]. The distributions of *P. uterobilateralis* and *P. africanus* strongly overlapped. Paragonimiasis caused by the species *P. uterobilateralis* is usually pulmonary [5].

#### Paragonimus gondwanensis

In 2014, *Paragonimus gondwanensis* was described from the lungs of naturally infected cats and civets in Cameroon by Bayssade-Dufour et al. [54]. This species is the most recently reported human-infecting *Paragonimus* species. The following year, Bayssade-Dufour et al. described another species, *P. kerberti*, but there are no reports of paragonimiasis in humans caused by this species [5]. There are few studies on this species, so more studies on its distribution and disease characterization are needed.

Human cases caused by *P. westermani* have been reported in China, Japan, the Republic of Korea, the Far East of Russia and the Philippines, whereas in other countries with this species’ range (e.g., Thailand, Malaysia), human cases caused by this species have not been reported. Given the taxonomy complexity and controversy of *Paragonimus* species, we will not discuss much about it in this study. The taxonomy we used in this article follows that of Blair et al. 1999, Yoshida et al. 2019, and Zhou et al. 2021 [2–4].

In recent years, many general reviews have focused on the biology, evolution and medical significance of *Paragonimus* or paragonimiasis [3, 5, 16, 55–59]. These studies cover many aspects and are highly valuable. However, very few studies have focused on mapping the occurences of *Paragonimus* species (OPSs) or paragonimiasis (only two studies in recent years): one focused on Africa [16], and the other focused on China [4]. However, these two studies are regional and not global. Mapping is essential for identifying endemic foci; e.g., a study revealed that infections of *Clonorchis sinensis* in animals are primarily concentrated in regions with low altitudes and high precipitation [60]. Thus, we aimed to (1) update the World Register of *Paragonimus* species, (2) map the distributions of OPSs and paragonimiasis cases globally, and (3) present a list of the second hosts of *Paragonimus* species. The list of the second hosts is included in this study because of its importance. This is not only because humans are usually infected with *Paragonimus* by ingesting crustaceans [2, 4, 56], but more importantly, isolating *Paragonimus* metacercariae carried by crustacean hosts is the most commonly used way in which epidemiologists and parasitologists obtain *Paragonimus* samples.

## Methods

### Search strategy

The search for target literature was conducted via four global and four regional databases: Google Scholar, Web of Science, PubMed, ScienceDirect, African Journals Online (Africa), Chinese National Knowledge Infrastructure (CNKI, https://www.cnki.net/, China), Wanfang Database (https://www.wanfangdata.com.cn/, China), and Chongqing VIP Chinese Science and Technology Journal Database (http://qikan.cqvip.com/, China). The search terms used were as follows: *Paragonimus*, lung fluke and paragonimiasis (see Additional file 1). The search terms in Chinese were used for the three Chinese databases, and the search terms in English were used for the other databases from 1 January, 1900 to 31 December, 2024.

### Inclusion and exclusion criteria

Studies of OPSs and paragonimiasis were included if they met the following criteria: (1) the OPSs were verified according to the study, (2) the site where the *Paragonimus* species occurred was clear, or (3) the case reports of patients with a confirmed diagnosis of paragonimiasis were included. Studies of OPSs that met the following criteria were excluded: (1) the OPSs were not confirmed (e.g., a diagnosis of paragonimiasis using ELISA only), (2) studies that reported the OPSs in a large area (e.g., a country or a large-area state or province), and (3) case reports that did not show where the patients ate crustaceans and got infected (Given inherent human mobility, we cannot reliably equate the location where paragonimiasis is diagnosed with the area where *Paragonimus* species are endemic). Publications of case reports were extracted for mapping the distribution of paragonimiasis cases.

### Data extraction and quality assessment

Eligible studies of OPSs were divided into three groups on the basis of the study region: Asia, the Americas, and Africa. The following data were extracted from the included studies: *Paragonimus* species, second hosts, site, longitude, latitude, publication year, decade, study language, and corresponding literature (see Additional file 2). Most studies do not provide information on the longitude and latitude of the OPSs, so we obtained this information via Google Maps (https://www.google.com/maps) based on the corresponding sites. “Decade” means the decade in which the study was published. Part of the data from China and Africa were obtained directly from the two studies mentioned above and another study focusing on China in 2000 [4, 5, 61]. The data of eligible studies of paragonimiasis cases can be seen in Additional file 3. The first author and the second author independently assessed the risk of bias for the included studies via the Joanna Briggs Institute (JBI) Critical Appraisal Tools [62]. “Low risk of bias” represents a summary score of 0–3, “moderate risk of bias” represents a summary score of 4–6, and “high risk of bias” represents a summary score of 7–10. Each study was assigned a low, moderate or high risk of bias.

The names of different second hosts recorded in the file are the scientific names used currently. Updated names of crustacean hosts were obtained via the WoRMS database (https://www.marinespecies.org/index.php). In some publications, the species names were wrongly used. Some could be easily identified (such as *Eriocheir japonica* wrongly used as *Eriocheir japonicus* and *Eriocher japonieum*), so that we can rectify these species names in Additional file 4. However, some species names cannot be related to any of the scientific names used now. These names are marked with asterisks.

### Data analysis and mapping

The data were organized and analyzed in Microsoft Excel (version 2016; Microsoft Corporation, Redmond, USA). Figures except for the maps and the life cycle figures were produced on the online platform “https://www.bioinformatics.com.cn”. The map data set is provided by Geospatial Data Cloud site, Computer Network Information Center, Chinese Academy of Sciences. (http://www.gscloud.cn) [63]. The maps of different time periods of *Paragonimus* species were produced via ArcGIS 10.2 (Environment System Research Institute, Redlands, USA), by importing the coordinate data to the software. The period was divided according to the estimation of paragonimiasis in 1995 by the WHO [64] and in 2005 and 2015 by the Global Burden of Disease Study [65]. The different periods of OPSs in the five maps were as follows: “total” (mapping all the OPSs), “before 1995” (mapping the OPSs before 1995), 1996–2005 (mapping the OPSs from 1996 to 2005), 2006–2015 (mapping the OPSs from 2006 to 2015), and 2016–2024 (mapping the OPSs from 2016 to 31-December, 2024). The coordinate information for each map is provided in Additional file 5.

## Results

### Study selection and data characteristics

For OPSs studies, a total of 19,854 articles were identified in eight databases. After title and abstract screening and duplicate removal, 17,611 articles were excluded. After full-text assessment, 931 of 2243 articles meeting the inclusion criteria were included. In all the included studies, those in English were most frequently used (approximately 48.7%). Chinese studies were the second most common, followed by Japanese, Spanish, Korean, French, and German studies (see Additional file 6). In the Americas, English studies were also the most frequently included, followed by Spanish studies (see Additional file 6). In Africa, English studies were most frequently included, followed by French and German studies (see Additional file 6). In contrast to studies in the Americas and Africa, studies in Chinese have the maximum quantity in Asia, followed by studies in English. Notably, 39 and 21 Japanese and Korean studies, respectively, were included (see Additional file 6).

**Fig. 2:**
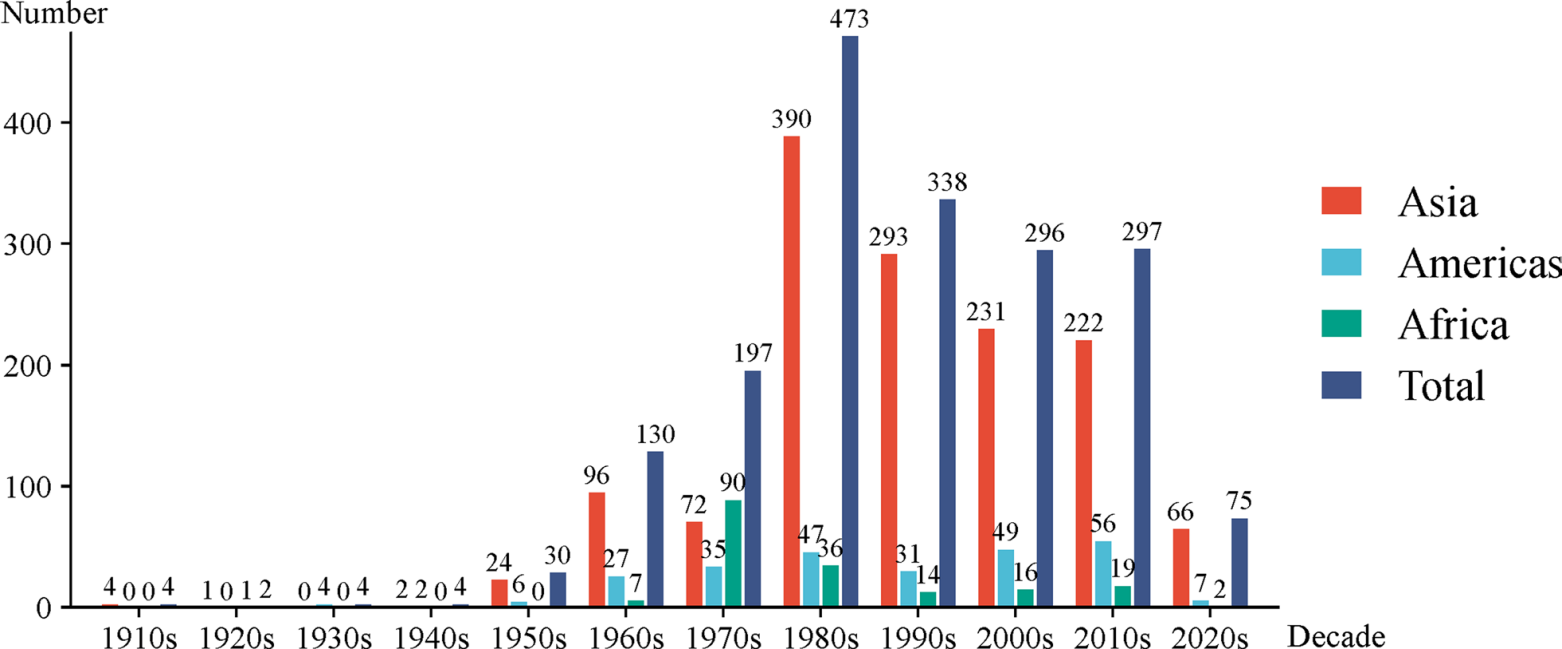
Bar chart showing OPSs (Occurrences of *Paragonimus* species) by decade (e.g. 1990s include the years of “1990 to 1999” in this bar chart) and region. Different colors indicate different regions. “Total” represents the sum of the three regions (Asia, the Americas and Africa)

Few studies recorded the OPSs before the 1950s. Since the 1950s, the number of OPSs has increased rapidly and peaked in the 1980s. Since the 1980s, the number of OPSs has declined gradually and remained stable in the 2000s and 2010s. In contrast to other regions, Asia experienced a decline in the 1970s. In nearly all decades, the number of OPSs in Asia reached a maximum, and that in Africa reached a minimum. Notably, in the 1970s, the number of OPSs in Africa (90) surpassed that in Asia (72) and reached a maximum (Fig. 2; see Additional file 7).

### Mapping of *Paragonimus* species

**Fig. 3:**
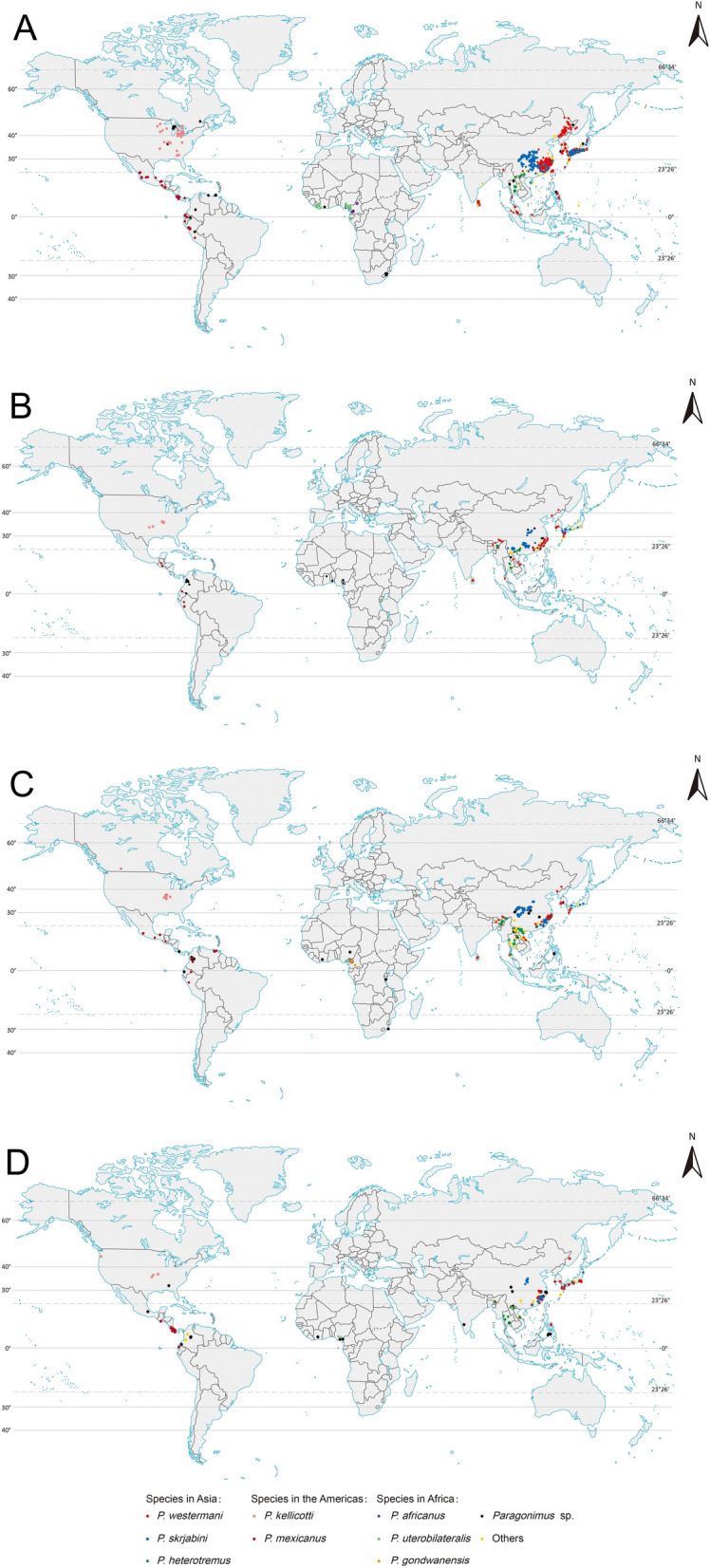
Maps of the OPSs (Occurrences of *Paragonimus* species) globally during different periods. **A**. (1900–1995), **B**. (1996–2005), **C**. (2006–2015), **D**. (2016–31-December, 2024). The dots of different colors indicate different *Paragonimus* species. Undetermined *Paragonimus* species were categorized as "*Paragonimu*s sp.", and those not infecting humans as "Others". The map data set is provided by Geospatial Data Cloud site, Computer Network Information Center, Chinese Academy of Sciences. (http://www.gscloud.cn). Map approval number: GS(2026)0525

We constructed five maps to visualize the OPSs, especially the eight species that infect humans (Fig. 3). Among the 1915 OPSs extracted from the included studies, the majority were reported in Asia (1466, 76.6%), followed by the Americas (264, 13.8%) and Africa (185, 9.6%). (see Additional file 5). Three human-infecting species are distributed in Asia (*P. westermani*, *P. skrjabini* and *P.* *heterotremus*), two human-infecting species (*P. kellicotti* and *P. mexicanus*) from the Americas and three from Africa (*P. africanus*, *P. uterobilateralis* and *P. gondwanensis*). This study suggests that *Paragonimus* species are distributed from 12° S to 50° N globally, except four “outliers” in South Africa (approximately 30° S). Notably, *Paragonimus* species in the Northern Hemisphere can extend to high-latitude regions, whereas those in the Southern Hemisphere can only live in tropical regions. The number of second hosts of *Paragonimus* species globally is highest in Asia, medium in the Americas and lowest in Africa (see Additional file 4). Additional file 4 was extracted from Additional file 2 to clearly display the second hosts from different regions.

### Asia

There are three human-infecting species distributed in Asia. *P. westermani* has the broadest distribution (East Asia, the Far East, Southeast Asia, India, Sri Lanka, probably Nepal, New Guinea) not only in Asia but also in the world. *P. skrjabini* is distributed mainly in China and Japan. *P. heterotremus* is limited to the Indochina Peninsula and southwestern border regions of China and northeastern India [66] (Table 1; Fig. 3).

China, where three Asian human-infecting species are present, has the greatest species richness of *Paragonimus* [2, 4]. *P. westermani* and *P. skrjabini* are the dominant species. *P. westermani* is distributed mainly in southeastern China and northeastern China, whereas *P. skrjabini* is distributed mainly in central China. In contrast to the two widely distributed species, *P. heterotremus* is limited to the southwestern border regions of China (Guangxi Zhuang autonomous region and Yunnan Province) [4] (Fig. 3). In Japan, there are two human-infecting species: *P. westermani* and *P. skrjabini miyazakii*. The distributions of the two species overlapped across nearly all of Japan except Hokkaido (Fig. 3). The occurrence of *P. ohirai* is also frequently reported in Japan [7, 67]. There is only one human-infecting species in the Republic of Korea: *P. westermani.* This species is distributed throughout the country. Articles on the Far East of Russia were so rare that only a few occurrences were mapped (Fig. 3). In this area, there is only one human-infecting species: *P. westermani ichunensis*. In India, there are three human-infecting species that are distributed mainly in northeastern regions: *P. westermani, P. skrjabini* and *P. heterotremus*. In Sri Lanka, there is only one human-infecting species: *P. westermani.* Furthermore, the situation is slightly different in Southeast Asia compared with that in other regions. In Malaysia, the Philippines and Indonesia, *P. westermani* is the dominant species without a distribution of *P. heterotremus*, whereas in other countries on the Indochina Peninsula, *P. heterotremus* is the dominant species (Table 1; Fig. 3).

The endemic foci of *Paragonimus* species in Asia have been stable since 1995 (Fig. 3). OPSs in northeastern China were reported mainly before 1995. After 1995, few occurrences were reported in this region (Fig. 3). After 2015, there was no occurrence in this region (Fig. 3D). Occurrences in the Indochina Peninsula from 2006 to 2015 are very intensive, even more than those in the period before 1995 (Fig. 3A, C).

In Asia, China has the greatest species richness of the second hosts of *Paragonimus* species (62), which is far greater than that in other regions of the world (see Additional file 4). The species richness of the second hosts of *Paragonimus* species in all other Asian countries is lower than 10. In northeastern China, the Republic of Korea and the Far East in Russia, freshwater crayfish are the second hosts of *Paragonimus* species, whereas freshwater crabs are the second hosts in other regions of Asia. More information on the second intermediate hosts of *Paragonimus* species in Asia can be found in Additional file 4.

### The Americas

There are two human-infecting *Paragonimus* species distributed in the Americas: *P. kellicotti* and *P. mexicanus*. The two species are entirely geographically separated: *P. kellicotti* is distributed in North America, whereas *P. mexicanus* is distributed in Latin America (Fig. 3). There is a “gap region” between the USA and Mexico, in which there are no reports of OPSs (Fig. 3).

In the USA and Canada, only one species has been reported: *P. kellicotti*. It is distributed mostly in the eastern part of the USA, and Ontario and Quebec in Canada (Fig. 3). A study published in 2020 reported the OPSs in canine feces at three off-leash dog parks in Portland, Oregon, USA, which is located on the Pacific coast and is far from the main endemic foci in the USA [68] (Fig. 3). Another study published in 2011 detected *P. kellicotti* from coyote feces in Calgary, Alberta, Canada, which is also far from the main endemic foci in Canada [69] (Fig. 3). In countries in Latin America, there is only one human-infecting species: *P. mexicanus*. In Mexico, the species is distributed in the southern part of the country. In Colombia, the species is distributed in the northwest of the country. In Venezuela, *P. mexicanus* is distributed in the northern coastal region. In Ecuador, the species is distributed in the western coastal region and northern part of the country. In Peru, the species is distributed in the western part (Fig. 3).

In the Americas, Mexico has the highest species richness of the second hosts (9), which is one more than that of Colombia. In the USA and Canada, freshwater crayfish are the second hosts of *Paragonimus* species as freshwater crabs do not occur there, whereas freshwater crabs are the second hosts of *Paragonimus* species in other regions of the Americas (Additional file 2; Additional file 4). More information on the second intermediate hosts of *Paragonimus* species in the Americas can be found in Additional file 4.

### Africa

Three human-infecting species have been reported in Africa: *P. africanus*, *P. uterobilateralis* and *P. gondwanensis*. The three species are mostly distributed in West and Central Africa. In Cameroon, all three human-infecting species are present and distributed mainly in the western coastal regions. In Gabon, only one species, *P. uterobilateralis*, which is distributed in the northwestern coastal regions, has been reported. In Liberia, only one species has been reported: *P. uterobilateralis*. In Nigeria, three human-infecting *Paragonimus* species have been reported, and they are distributed mainly in the southern coastal regions. Occurrences of *P. uterobilateralis* and *Paragonimus* species undetermined were reported in Tanzania and South Africa, respectively (Fig. 3).

In Africa, Cameroon has the greatest species richness (5) of second hosts, with one more than that of Nigeria (see Additional file 4). There are no freshwater crayfish species that serve as second hosts of *Paragonimus* species in Africa, unlike in Asia and the Americas (see Additional file 4). More information on the second intermediate hosts of *Paragonimus* species in Africa can be found in Additional file 4.

### Mapping of paragonimiasis case distribution

To further analyze the relationship between the distribution of *Paragonimus* species and the prevalence of paragonimiasis from a public health perspective, we searched relevant literature and created a global map of paragonimiasis case distribution. In total, 666 papers were included in the data set, numbering 6561 cases. To date, according to our study, cases of paragonimiasis have been reported in 35 countries (Additional file 3). Generally, the ranking of paragonimiasis case reports by number is Asia, the Americas, Africa, Europe, and Oceania (from high to low). China reported the highest number of paragonimiasis cases (*n* = 6047). With the exception of China, no other country reported over 200 cases, indicating a global imbalance in the reporting of paragonimiasis cases. One potential explanation is the difference in research attention devoted to *Paragonimus* studies across nations. In some countries including Malaysia (number of paragonimiasis cases = 1), Vietnam (1) and Colombia (3), OPSs were reported frequently, whereas only a small number of paragonimiasis cases were documented. In contrast, in countries such as Saudi Arabia (1), Denmark (1), Switzerland (1), Australia (1), New Zealand (1), Spain (1), England (3), and France (4), no OPSs were detected, yet paragonimiasis cases were reported. It may be attributed to imported cases from other paragonimiasis-endemic foci, highlighting the need for vigilance against biological invasion by these parasites.

## Discussion

This study comprehensively investigated the global distribution of *Paragonimus* on the basis of literature reports. For some species with disputed names, we did not conduct further species identification, including the cryptic species within the complex species [5, 70]. We hope to provide public health workers with more reliable evidence for the prevention and control of paragonimiasis from a global perspective.

A comparison of different periods revealed that there was an “outbreak period” of reports of new *Paragonimus* species (32 species) in the 1960s and 1970s (Table 1). In contrast, since the start of the twenty-first century, only five new species were reported. The potential reasons may include the following: fewer as-yet-unknown cryptic *Paragonimus* species than previously; and diminished research interest among investigators.

Due to the complexity of *Paragonimus* life cycle, from the perspective of public health researchers, strategies for preventing and controlling paragonimiasis should not only focus on the human population but also pay attention to the hosts of *Paragonimus*, especially the crustacean hosts. Different epidemic areas should develop targeted prevention strategies due to variations in intermediate hosts and dietary habits. Notably, the invasive species “*Procambarus clarkii*”, which is native to North America, has been reported to harbor *Paragonimus* species in Asia and South America [71–73]. In particular, “*Procambarus clarkii*” is cooked as food and is very popular in Asia. The occurrence of biological invasions and species extinction may alter the burden of paragonimiasis. Attention should be paid to such invasions in endemic foci because it may increase the burden of paragonimiasis.

Based on the global distribution of *Paragonimus* species, *Paragonimus* has a dominant distribution in the Northern Hemisphere between 0°N and 50°N, in tropical and subtropical regions. In Africa, *Paragonimus* species are distributed mainly in West Africa and Central Africa, where the tropical rainforest climate is common, with a hot climate and sufficient precipitation. Other regions in Africa that lack precipitation are mainly deserts and grasslands, which may not be suitable habitats for *Paragonimus* species and their first and second hosts. Notably, some regions in Australia in which freshwater snails and crabs are distributed [74, 75] are suitable habitats for *Paragonimus* species; however, no occurrences of the species have been reported in these regions. Specifically, some countries with warm or hot climates and sufficient precipitation, such as India, Cambodia, Myanmar and Brazil, are suitable habitats for *Paragonimus* species. However, there are very few or even no reports of the OPSs in these countries. In contrast, a certain number of cases were reported in these countries or regions. Therefore, there is a speculation here that the occurrences of the *Paragonimus* species identified via mapping may have been underestimated. Studies in many countries have not been performed enough. The most direct evidence is that there have been no reports of the OPSs in Cambodia, around which countries all have endemic foci of paragonimiasis. In Myanmar, there is one report of the occurrence of *P. heterotremus* [76]. The situations of many other countries are similar to those of Cambodia and Myanmar, especially countries with humid climates in tropical and subtropical regions. More attention should be paid to these countries, as there may be new species that have never been found in these countries.

Figures 2 and 3 show that the number of reports of *Paragonimus* species has decreased over the past 40 years. This may have resulted from control measures implemented by governments, economic growth, improvements in public health awareness, decreased research interest, and so on. In the 1970s, the number of OPSs in Africa surpassed that in Asia (Fig. 2), which is abnormal. This may be attributed to food shortages and simultaneous consumption of freshwater crabs resulting from the Nigerian Civil War of 1967–1970 [77–79]. Crabs are more likely to be consumed by people living in rural regions due to poverty and food shortages. While this practice has become rare today, it is crucial to remain aware that it may resurface. Such a resurgence could be triggered by factors like infectious diseases, wars, or other crises that lead to regional food shortages.

**Fig. 4:**
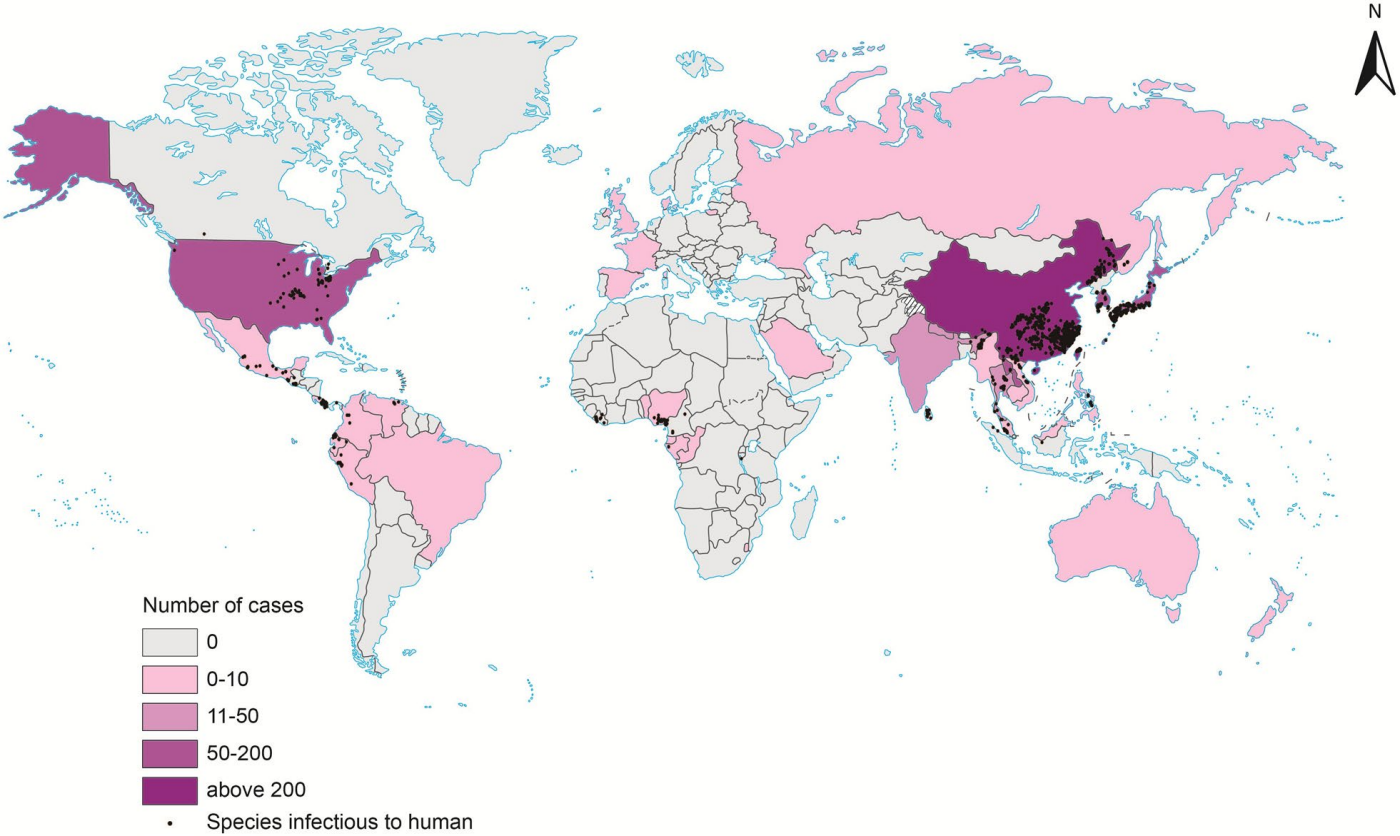
Global map showing the number of paragonimiasis per country, from reported cases. The purpler colors indicate a higher number of paragonimiasis case reports, while pinker shades indicate lower number. White indicates no case reports in the country. The black dots indicate the exact location of human-infecting *Paragonimus* species. The map data set is provided by Geospatial Data Cloud site, Computer Network Information Center, Chinese Academy of Sciences. (http://www.gscloud.cn). Map approval number: GS(2026)0525

Based on paragonimiasis case reports mapping (Fig. 4), in countries, such as Australia, some North Africa countries and European countries (Fig. 4), no *Paragonimus* species were reported, while paragonimiasis cases were documented. This phenomenon may be attributed to the global population mobility driven by enhanced transportation accessibility. In this era of advanced development, we should remain vigilant against the potential establishment of *Paragonimus* and other endemic parasitic organisms in previously non-endemic regions via population mobility.

Importantly, we are trying to collect more data to present the authentic “*Paragonimus* world”. However, our study only reflects the distribution of *Paragonimus* species and cases caused by them based on these literatures we compiled, and is by no means an authentic distribution. How much data we can compile depends on the interest of researchers, surveillance and reporting biases, literature accessibility, and so on.

## Conclusions

Our study updated the world register of *Paragonimus* species and mapped their global distribution. On the basis of these findings, *Paragonimus* species and paragonimiasis cases exhibit an imbalanced distribution across hemispheres, continents, and countries. The prevalence of *Paragonimus* species in many regions and countries may be underestimated. It is recommended that more epidemiological surveys or other surveys on *Paragonimus* species be conducted in countries or regions suitable for the survival of *Paragonimus* species but with relatively few or no reported occurrences. This study enhances the understanding of the spatiotemporal distribution of *Paragonimus* species worldwide and can provide support for the intervention and control of paragonimiasis (lung fluke disease), thereby promoting the improvement of public health.

## Supplementary Information

Supplementary Material 1. Table S1. Search terms for literature in eight databases

Supplementary Material 2. Data recording OPSs extracted from eligible studies

Supplementary Material 3. Data recording paragonimiasis case reports

Supplementary Material 4. The list of second intermediate hosts of *Paragonimus*

Supplementary Material 5. Coordinates of OPSs for the maps

Supplementary Material 6. The number of studies by different languages

Supplementary Material 7. The number of OPSs by decade

## Data Availability

All data generated or analyzed during this study are included in this published article and its additional information files.

## Abbreviations

OPSs: Occurences of *Paragonimus* species
WHO: World Health Organization
ELISA: Enzyme-linked-immunosorbant assay
USA: The United States of America

## References

[1] Daumerie D, Savioli L, Peters P. Working to overcome the global impact of neglected tropical diseases: first WHO report on neglected tropical diseases. Geneva: World Health Organization; 2010.

[2] Blair D, Xu ZB, Agatsuma T. Paragonimiasis and the genus *Paragonimus*. Adv Parasitol. 1999;42:113–222. 10050273 10.1016/s0065-308x(08)60149-9

[3] Yoshida A, Doanh PN, Maruyama H. *Paragonimus* and paragonimiasis in Asia: an update. Acta Trop. 2019;199:105074. 31295431 10.1016/j.actatropica.2019.105074

[4] Zhou XJ, Yang Q, Tan QH, Zhang LY, Shi LB, Zou JX. *Paragonimus* and its hosts in China: an update. Acta Trop. 2021;223:106094. 34389330 10.1016/j.actatropica.2021.106094

[5] Blair D. Lung flukes of the genus *Paragonimus*: ancient and re-emerging pathogens. Parasitology. 2022;149(10):1286–95. 35292126 10.1017/S0031182022000300 PMC10090773

[6] Fürst T, Keiser J, Utzinger J. Global burden of human food-borne trematodiasis: a systematic review and meta-analysis. Lancet Infect Dis. 2012;12(3):210–21. 22108757 10.1016/S1473-3099(11)70294-8

[7] Yokogawa M. *Paragonimus* and paragonimiasis. Adv Parasitol. 1969;7:375–87. 4935271 10.1016/s0065-308x(08)60440-6

[8] Miyazaki I, Hirose H. Immature lung flukes first found in the muscle of the wild boar in Japan. J Parasitol. 1976;62(5):836–7. 978373

[9] Kirino Y, Nakano N, Hagio M, Hidaka Y, Nakamura-Uchiyama F, Nawa Y, et al. Infection of a group of boar-hunting dogs with *Paragonimus westermani* in Miyazaki Prefecture, Japan. Vet Parasitol. 2008;158(4):376–9. 18976862 10.1016/j.vetpar.2008.09.017

[10] Sugiyama H, Shibata K, Kawakami Y, Arakawa K, Morishima Y, Yamasaki H, et al. Paragonimiasis due to the consumption of wild boar meat in Japan: contamination levels of lung fluke larvae in muscle samples of wild boars caught in Kagoshima Prefecture. Jpn J Infect Dis. 2015;68(6):536–7. 26370424 10.7883/yoken.JJID.2015.280

[11] Xue HB, Zhang DR. Report and analysis of cases of paragonimiasis caused by consuming drunk crabs. Parasitos Infect Dis. 1999;01:38 (in Chinese).

[12] Kim SY, Park SJ, Bae SY, et al. A case of subcutaneous paragonimiasis presented with pleural effusion. Clin Med Pediatrics. 2008;51(7):760–5.

[13] Cho AR, Lee HR, Lee KS, Cho YK, Kim CJ, Woo YJ, et al. A case of pulmonary paragonimiasis with involvement of the abdominal muscle in a 9-year-old girl. Korean J Parasitol. 2011;49(4):409. 22355209 10.3347/kjp.2011.49.4.409 PMC3279680

[14] Xu H, Hong XF. A case of paragonimiasis caused by eating drunk crabs. J Cardiovasc Pulm Dis. 2019;38(03):312–20 (in Chinese).

[15] Traoré SG, Odermatt P, Bonfoh B, Utzinger J, Aka ND, Adoubryn KD, et al. No *Paragonimus* in high-risk groups in Côte d’Ivoire, but considerable prevalence of helminths and intestinal protozoon infections. Parasit Vectors. 2011;4:1–10. 21639877 10.1186/1756-3305-4-96 PMC3130684

[16] Cumberlidge N, Rollinson D, Vercruysse J, Tchuenté LAT, Webster B, Clark PF. *Paragonimus* and paragonimiasis in West and Central Africa: unresolved questions. Parasitology. 2018;145(13):1748–57. 30210013 10.1017/S0031182018001439

[17] Hirose MH. Immature lung flukes first found in the muscle of the wild boar in Japan. J Parasitol. 1976;62(5):836–7. 978373

[18] Singh TS, Sugiyama H, Umehara A, Hiese S, Khalo K. *Paragonimus heterotremus* infection in Nagaland: a new focus of paragonimiasis in India. Indian J Med Microbiol. 2009;27(2):123–7. 19384034 10.4103/0255-0857.49424

[19] Blair D, Chang ZS, Chen MG, Cui AL, Wu B, Agatsuma T, et al. *Paragonimus skrjabini* Chen, 1959 (Digenea: Paragonimidae) and related species in eastern Asia: a combined molecular and morphological approach to identification and taxonomy. Syst Parasitol. 2005;60:1–21. 15791397 10.1007/s11230-004-1378-5

[20] Devi KR, Narain K, Mahanta J, Nirmolia T, Blair D, Saikia SP, et al. Presence of three distinct genotypes within the *Paragonimus westermani* complex in northeastern India. Parasitology. 2013;140(1):76–86. 22917216 10.1017/S0031182012001229

[21] Hernández-chea R, Jiménez-rocha AE, Castro R, Blair D, Dolz G. Morphological and molecular characterization of the metacercaria of *Paragonimus caliensis*, as a separate species from *P. mexicanus* in Costa Rica. Parasitol Int. 2017;66(2):126–33. 28027969 10.1016/j.parint.2016.12.006

[22] Liu SC, Zhang DC, Li YS, Xu ZH. Discovery of mixed infection of *Euparagonimus cenocopiosls* and *Paragonimus westermani* and comparative study on their life history. Chin Sci Bull. 1981;03:186–8 (in Chinese).

[23] Kerbert C. Zur Trematoden-Kenntnis. Zool Anz. 1878;1(1):271G3.

[24] Procop GW. North American paragonimiasis (caused by *Paragonimus kellicotti*) in the context of global paragonimiasis. Clin Microbiol Rev. 2009;22(3):415–46. 19597007 10.1128/CMR.00005-08 PMC2708389

[25] Gulati A. On the occurrence of a lung fluke *Paragonimus edwardsi* n. sp. in a Palm Civet (*Paradoxurus grayi*) in Kumaon Hills. Mem Entomol Soc India. 1926;3:187–90.

[26] Miyazaki I. *Paragonimus filipinus* sp. N. found in Leyte the Republic of the Philippines (Trematoda: Troglotrematidae). Jpn J Parasitol. 1978;5:5–10.

[27] Li YS, Cheng YZ. Distinguishing the Independence of several *Paragonimus* species in China. Wuyi Sci J. 1992;00:269–76 (in Chinese).

[28] Blair D, Agatsuma T, Watanobe T, Okamoto M, Ito A. Geographical genetic structure within the human lung fluke, *Paragonimus westermani*, detected from DNA sequences. Parasitology. 1997;115(4):411–7. 9364568 10.1017/s0031182097001534

[29] Zhang YJ, Shen YP, Shi ZM, Zhang ZH, Feng XC, Yu XC. A study on *Paragonimus asymmetricus*. Parasitos Infect Dis. 1997;04:147–50 (in Chinese).

[30] Sato Y, Iwagami M, Iwashita J, Yukawa M, Blas BL, et al. Phylogenetic status of a lung fluke in the Philippines based on mitochondrial genome. Jpn J Trop Med Hyg. 2003;31(1):1–3.

[31] Chung HL, Ts’ao WC. *Paragonimus westermani* (Szechuan variety) and a new species of lung fluke-*Paragonimus szechuanensis*. Part I. studies on morphology and life history of *Paragonimus szechuanensis*. Chin Med J. 1962;81(06):354–78. 13879399

[32] Chung HL, Ts’ao WC, Liu TH, Chou KH, Yang CY, Fu LH, et al. *Paragonimus westermani* (Szechuan variety) and a new species of lung fluke-*Paragonimus szechuanensis*. part II studies on clinical aspects of *Paragonimiasis szechuanensis*-a new clinical entity. Chin Med J. 1962;81(07):419–34. 13879399

[33] Chen XT. Some issues to be noted in the research of newly discovered *Paragonimus* species and study on paragonimids in China. Acta Sci Nat Univ Sunyatseni. 1962;03:58–64 (in Chinese).

[34] Cui AL, Chang ZS, Chen MG, Blair D, Chen SH, Zhang YN, et al. Taxonomic status of *P**aragonimus skrjabini* populations from five provinces in China in morphology. Chi J Anim Infect Dis. 2003;01:4–7 (in Chinese).

[35] Cui AL, Chang ZS, Chen MG, Blair D, Chen SH, Zhang YN, et al. Study on DNA sequences of *Paragonimus **skrjabini* populations from five provinces in China. Chi J Parasitol Parasitic Dis. 2003;02:9–13 (in Chinese). 12884612

[36] Chung HL, Hsu CP, Ho LY, Kao PC, Shao L, Chiu FH, et al. Studies on a new pathogenic lung fluke-*Paragonimus hueitungensis* sp. nov. Chi Med J. 1977;3(06):379–94. 414893

[37] Zhang SY. Investigation on epidemic focus of paragonimiasis in Sangyan district Fujian province and study on the independence of *Pagumogonimus veocularis*. Fujian: Fujian Medical University; 2005. (in Chinese).

[38] Chen XT, Xia DG. Preliminary report on a new species of *Paragonimus*. Acta Sci Nat Univ Sun Yat Sen. 1964;02:236–8+84-86 (in Chinese).

[39] Chung HL, Ho LY, Cheng LT, Tsao WC. The discovery in Yunnan province of 2 new species of lung flukes-*Paragonimus tuanshanensis* sp. nov. and *Paragonimus menglaensis* sp. nov. part I. studies on morphology and life history with discussion on possible pathogenicity to man. Chin Med J. 1964;83(10):641–59. 14228262

[40] Ch’en HT. *Paragonimus*, *Pagumogonimus* and a *Paragonimus*-like trematode in man. Chin Med J. 1965;84(12):781–91. 5864046

[41] Ward HB. On the presence of *Distoma westermanni* in the United States. Vet Mag. 1894:355–357.

[42] Kellicott DS. Certain entozoa of the dog and sheep. Trans Ohio State Med Soc. 1894:122–130.

[43] Ward HB. Data for the determination of human Entozoa. II. Trans Am Microsc Soc. 1908;28:177–201.

[44] Ward HB, Hirsch EF. The species of *Paragonimus* and their differentiation. Ann Trop Med Parasitol. 1915;9(1):109–62.

[45] Stuht JN, Youatt WG. Heartworms and lung flukes from red foxes in Michigan. J Wildl Manage. 1972. 10.2307/3799205.

[46] Miyazaki I, Ishii Y. Studies on the Mexican lung flukes, with special reference to a description of *Paragonimus mexicanus* sp. nov.(Trematoda: Troglotrematidae). Jpn J Parasitol. 1968;17:445–53.

[47] Miyazaki I, Kifune T, Lamothe-argumedo R. Taxonomical and biological studies on the lung flukes of Central America. Occasional Publications of the Department of Parasitology, School of Medicine. Fukuoka: Fukuoka University; 1980.

[48] López-Caballero J, Oceguera-Figueroa A, León-Règagnon V. Detection of multiple species of human *Paragonimus* from Mexico using morphological data and molecular barcodes. Mol Ecol Resour. 2013;13:1125–36. 23530893 10.1111/1755-0998.12093

[49] Tongu Y. The species of *Paragonimus* in Latin America. Summary of Health Care Department of School of Medicine at Okayama University. 2001;12(1):1–5.

[50] Lenis C, Galiano A, Vélez I, Vélez ID, Muskus C, Marcilla A. Morphological and molecular characterization of *Paragonimus caliensis* Little, 1968 (Trematoda: Paragonimidae) from Medellin and Pichinde, Colombia. Acta Trop. 2018;183:95–102. 29596790 10.1016/j.actatropica.2018.03.024

[51] Landaverde-gonzález P, Osgood J, Quiñonez CAM, Monzón V, Rodas A, Monroy C. The effect of landscape and human settlement on the genetic differentiation and presence of *Paragonimus* species in Mesoamerica. Int J Parasitol. 2022;52(1):13–21. 34371019 10.1016/j.ijpara.2021.05.010

[52] Voelker J, Vogel H. Two new species of *Paragonimus* in West Africa: *Paragonimus africanus* and *P. uterobilateralis*. Z Tropenmed Parasitol. 1965;16(2):125–48. 4954791

[53] Rabone M, Wiethase J, Clark PF, Rollinson D, Cumberlidge N, Emery AM. Endemicity of *Paragonimus* and paragonimiasis in sub-Saharan Africa: a systematic review and mapping reveals stability of transmission in endemic foci for a multi-host parasite system. PLoS Negl Trop Dis. 2021;15(2):e0009120. 33544705 10.1371/journal.pntd.0009120 PMC7891758

[54] Bayssade-dufour C, Chermette R, Šundić D, Radujković BM. *Paragonimus gondwanensis* n. sp.(Digenea, Paragonimidae), parasite of mammals (humans and carnivores) in Cameroon. Ecol Montenegrina. 2014;1(4):256–67.

[55] Adams AM. Foodborne trematodes: *Paragonimus* and *Fasciola*. In: Ortega Y, Sterling C, editors. Foodborne Parasites. USA: Springer; 2018. p. 293–324.

[56] Blair D. Paragonimiasis. In: Toledo R, Fried B, editors. Digenetic Trematodes. USA: Springer; 2019. p. 105–38.

[57] Chai JY, Jung BK. Epidemiology of trematode infections: an update. In: Toledo R, Fried B, editors. Digenetic Trematodes. USA: Springer. Springer International Publishing; 2019. p. 359–409. 10.1007/978-3-030-18616-6_12 31297768

[58] Coogle B, Sosland S, Bahr NC. A clinical review of human disease due to *Paragonimus kellicotti* in North America. Parasitology. 2022;149(10):1327–33. 35965058 10.1017/S0031182021001359 PMC9415338

[59] Tenorio JCB, Molina EC. Monsters in our food: foodborne trematodiasis in the Philippines and beyond. Vet Integr Sci. 2021;19(3):467–85.

[60] Liu K, Tan J, Xiao L, Pan RT, Yao XY, Shi FY, et al. Spatio-temporal disparities of *Clonorchis sinensis* infection in animal hosts in China: a systematic review and meta-analysis. Infect Dis Poverty. 2023;12(05):1–31. 37845775 10.1186/s40249-023-01146-4 PMC10580589

[61] Yang JS, Chen MG, Feng Z, Blair D. *Paragonimus* and paragonimiasis in China: a review of the literature. Chin J Parasitol Parasit Dis. 2000;2000:1–78.

[62] Munn Z, Moola S, Riitano D, Lisy K. The development of a critical appraisal tool for use in systematic reviews addressing questions of prevalence. Int J Health Policy Manag. 2014;3(3):123. 25197676 10.15171/ijhpm.2014.71 PMC4154549

[63] Geospatial Data Cloud site. http://www.gscloud.cn. Accessed 21 Apr 2025.

[64] WHO. Control of foodborne trematode infections: report of a WHO study group. Geneva: World Health Organization; 2001. 7740791

[65] Vos T, Allen C, Arora M, Barber RM, Bhutta ZA, Brown A, et al. Global, regional, and national incidence, prevalence, and years lived with disability for 310 diseases and injuries, 1990–2015: a systematic analysis for the Global Burden of Disease Study 2015. Lancet. 2016;388(10053):1545–602. 27733282 10.1016/S0140-6736(16)31678-6 PMC5055577

[66] Devi KR, Narain K, Bhattacharya S, Negmu K, Agatsuma T, Blair D, et al. Pleuropulmonary paragonimiasis due to *Paragonimus heterotremus*: molecular diagnosis, prevalence of infection and clinicoradiological features in an endemic area of northeastern India. Trans R Soc Trop Med Hyg. 2007;101(8):786–92. 17467757 10.1016/j.trstmh.2007.02.028

[67] Kawashima K, Miyahara M. Studies on *Paragonimus ohirai* Miyazaki, 1939, found in Yakushima, Kagoshima Prefecture, Japan. Jpn J Parasitol. 1974;23(6):369–75.

[68] Bishop GT, Debess E. Detection of parasites in canine feces at three off-leash dog parks in Portland, Oregon 2014. Vet Parasitol. 2020;22:100494. 10.1016/j.vprsr.2020.100494 33308738

[69] Watts AG, Alexander SM. Community variation of gastrointestinal parasites found in urban and rural coyotes (*Canis latrans*) of Calgary, Alberta. Cities Environ. 2012;4(1):11.

[70] Voronova AN, Vainutis KS, Tabakaeva TV, Sapotsky MV, Kakareka NN, Volkov YG, et al. Molecular identification of the trematode *P. ichunensis* stat. n. from lungs of siberian tigers justified reappraisal of *Paragonimus westermani* species complex. J Parasit Dis. 2022;46(3):744–53. 36091260 10.1007/s12639-022-01481-7 PMC9458828

[71] Yokogawa M. Studies on the biological aspects of the larval stages of *Paragonimus westermanii*, especially the invasion of the second intermediate hosts (I). Jpn J Med Sci Biol. 1952;5(4):221–37. 13010924 10.7883/yoken1952.5.221

[72] Yokogawa M. Studies on the biological aspects of the larval stages of *Paragonimus westermanii*, especially the invasion to the second intermediate hosts (III). Jpn J Med Sci Biol. 1953;6(2):107–17. 13096196 10.7883/yoken1952.6.107

[73] Phillips G, Hudson DM, Chaparro-Gutiérrez JJ. Presence of *Paragonimus* species within secondary crustacean hosts in Bogotá, Colombia. Revista Colombiana de Ciencias Pecuarias. 2019;32(2):150–7.

[74] Ponder WF, Colgan DJ. What makes a narrow-range taxon? Insights from Australian freshwater snails. Invertebr Syst. 2002;16(4):571–82.

[75] Yeo DCJ, Ng PKL, Cumberlidge N, Magalhães C, Daniels SR, Campos M. A global assessment of freshwater crab diversity (Crustacea: Decapoda: Brachyura). In: Balian EV, Lévequè C, Segers H, Martens M, editors, Freshwater Animal Diversity Assessment. Hydrobiologia. 2008. p. 275–286.

[76] Sanpool O, Intapan PM, Thanchomnang T, et al. Molecular variation in the *Paragonimus heterotremus* complex in Thailand and Myanmar. Korean J Parasitol. 2013;51(6):677. 24516273 10.3347/kjp.2013.51.6.677 PMC3916457

[77] Nwokolo C. Outbreak of paragonimiasis in Eastern Nigeria. Lancet. 1972;299(7740):32–3. 10.1016/s0140-6736(72)90017-7 4108826

[78] Nwokolo C. Endemic paragonimiasis in Eastern Nigeria. Clinical features and epidemiology of the recent outbreak following the Nigerian civil war. Trop Geogr Med. 1972;24(2):138–47. 5037686

[79] Nwokolo C. Endemic paragonimiasis in Africa. Bull World Health Organ. 1974;50(6):569. 4549201 PMC2481166

